# Supplementary material for: SERPIN-Derived Small Peptide (SP16) as a Potential Therapeutic Agent against HIV-Induced Inflammatory Molecules and Viral Replication in Cells of the Central Nervous System
Source: Cells. 2023 Feb 15;12(4):632. doi: 10.3390/cells12040632 (PMC9954444; doi:10.3390/cells12040632)
Supplement: Supplementary file 1 [file cells-12-00632-s001.zip › cells-2065100-supplementary.pdf]

SUPPLEMENTAL DATA

Article

SERPIN-Derived Small Peptide (SP16) as a Potential Therapeutic Agent against HIV-Induced Inflammatory Molecules and Viral Replication in Cells of the Central Nervous System

Yemmy Soler [1,2], Myosotys Rodriguez [1], Dana Austin [3], Cyrille Gineste [3], Cohava Gelber [3] and Nazira El-Hage [1,*]

[1] Department of Immunology and Nanomedicine, Herbert Wertheim College of Medicine, Miami, FL 33199, USA
[2] Department of Chemistry and Biochemistry, Florida International University, Miami, FL 33199, USA
[3] Serpin Pharma, 9501 Discovery Blvd Suite 120, Manassas, VA 20109, USA
* Correspondence: nelhage@fiu.edu; Tel.: +1-(305)-348-4346; Fax: +1-(305)-348-1109

Result

S1-Bioavailability of SP16 in mouse brain

Here, we confirmed that SP16 can be delivered to the brain after intranasal administration. Adult C57BL/6J mice were administered 10 μg/kg of SP16 at 20μL volume, in each nostril. After 24 hours, animals were sacrificed, and homogenized brains were used to extract SP16 in 200ml of acetonitrile containing Oxazepam-D5 and subsequently quantified by high-performance LC/MS-MS. About 125 nmol/L of SP16 (or 40% of the original concentration) was detected in brain homogenates (green arrow in panel A and C). Untreated animals are indicated with the yellow arrow (shown in panel B). Overall findings confirmed successful brain delivery of SP16 via intranasal delivery.

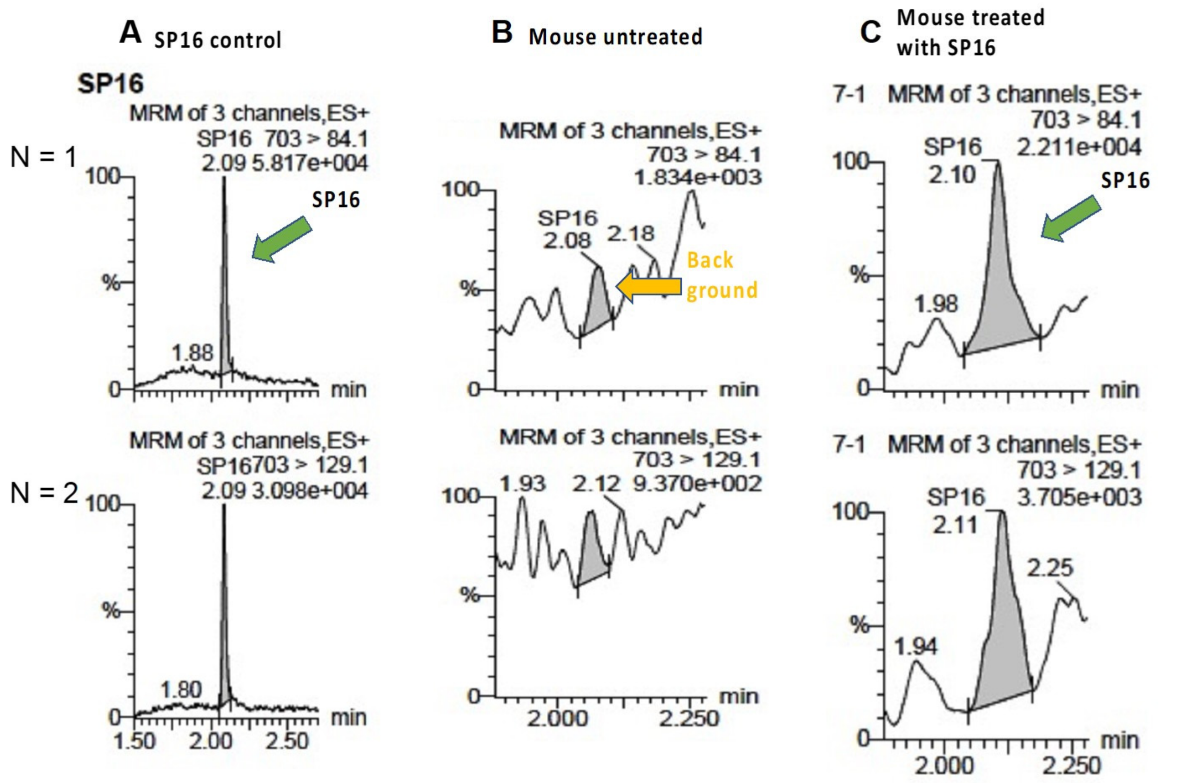

Supplementary figure S1. Bio-distribution of SP16 in EcoHIV infected brain after intranasal delivery. LC-MS/MS analysis of (A) SP16 in acetonitrile, (B) mouse untreated, (C) mouse treated intranasally with SP16 (C). This experiment was repeated in two adult mice (N=2), represented by the top and the bottom panel, respectively.

S2-Expression of LRP-1 in EcoHIV infected brain tissues

After treatments mice were sacrificed, and postmortem brain tissues were used to detect LRP-1. As shown in panel A, sliced tissues were co-labeled with antibody against LRP-1 (red) and MAP-2 (green), the marker for neurons. In panel B, tissues were labeled with LRP-1 (red) and GFAP (green), the marker for astrocytes. Cells were imaged using an inverted fluorescence microscope.

The fluorescence intensity for each antibody was measured using the Zen software (Zeiss, Germany). The respective numbers are represented in a graph bar, next to each panel.

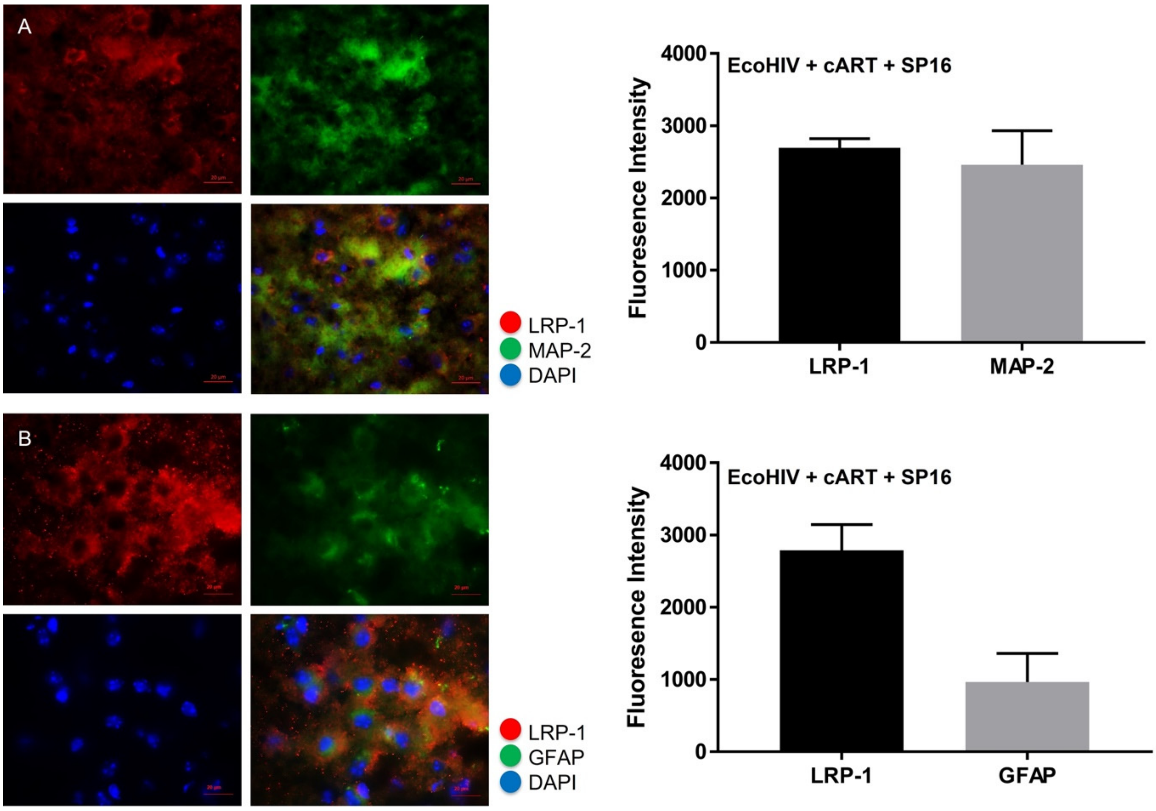

Supplementary figure S2. Expression of LRP-1 in EcoHIV infected brain. Brain tissue slices at 15 µM were labeled with antibody against (A) LRP-1 (red) and the neuronal marker, Microtubule-Associated Protein 2 (MAP-2, green) and (B) LRP-1 (red) and the astrocytes marker, Glial fibrillary acidic protein (GFAP, green). DAPI was used for nuclei staining (blue). The images were acquired using an inverted fluorescence microscope and the relative fluorescence units (RFU) were acquired using the Zen software (Zeiss). Scale Bar = 20 µM
